# Erfolgreiche Behandlung einer palmoplantaren Pustulose mit topischem Ruxolitinib

**DOI:** 10.1111/ddg.15854_g

**Published:** 2025-12-11

**Authors:** Neda Cramer, Phoebe Wellmann, Michael P. Schön, Rotraut Mössner

**Affiliations:** ^1^ Klinik für Dermatologie Venerologie and Allergologie Universitätsmedizin Göttingen

**Keywords:** JAK‐Inhibitoren, palmoplantare Pustulose, Psoriasis, JAK inhibitors, palmoplantar pustulosis, psoriasis

Sehr geehrte Herausgeber,

Die palmoplantare Pustulose (PPP) ist eine chronisch‐entzündliche Hautkrankheit, die als Variante der pustulösen Psoriasis gilt und durch rezidivierende sterile Pusteln, Erytheme und Schuppung an Handflächen und Fußsohlen charakterisiert ist.^1^ Aufgrund ihres chronischen und schubförmigen Verlaufs und der damit verbundenen Schmerzen kann sie zu funktionellen Behinderungen führen und die Lebensqualität der Patienten beeinträchtigen.^2^ Eine Differenzialdiagnose zur PPP ist die Psoriasis cum pustulatione, bei der Pusteln innerhalb oder am Rande der psoriatischen Plaques auftreten.^1^

Die Behandlung der PPP ist herausfordernd. Bislang gibt es keine therapeutischen Standards oder veröffentlichten Leitlinien. Zu den Behandlungsoptionen gehören topische Kortikosteroide, Phototherapie, systemische Retinoide und andere systemische Medikamente, die für Plaque‐Psoriasis oder Psoriasis‐Arthritis zugelassen sind, wie Methotrexat, Ciclosporin und Biologika.^3^, ^4^ Ferner gibt es zunehmend Evidenz aus Fallberichten und Fallserien, die auf eine ausgezeichnete Wirksamkeit von oral verabreichten Januskinase‐Inhibitoren (JAKi) bei der PPP hindeuten.^5^, ^6^, ^7^ Soweit uns bekannt, wurden bislang keine Berichte über eine topische Therapie mit JAKi veröffentlicht. Wir berichten über eine Patientin mit PPP, die therapieresistent gegenüber topischer Behandlung und UV‐Therapie war und erfolgreich *off‐label* mit topischem Ruxolitinib, einem selektiven JAK1/2‐Inhibitor, behandelt wurde.

Eine 66‐jährige Frau stellte sich mit seit 10 Monaten bestehenden juckenden palmoplantaren Hautveränderungen mit Erythem, Schuppung und Pusteln vor, wobei Pusteln auch in Bereichen ohne psoriatische Plaques sichtbar waren (Abbildung 1a). Wir stellten die Diagnose einer PPP. Die topischen Kortikosteroide Mometasonfuroat und Betamethasonvalerat unter Okklusion wurden seit Krankheitsbeginn angewendet, zeigten jedoch keine ausreichende therapeutische Wirkung, ebenso wenig wie die Phototherapie mit Psoralen plus Ultraviolett A (PUVA). Die Patientin war Raucherin mit 20 *pack‐years*. Die Familienanamnese war unauffällig. Des Weiteren bestand eine milde seronegative rheumatoide Arthritis der kleinen Finger‐ und Zehengelenke bei unauffälligem C‐reaktivem Protein, die bei Bedarf mit Ibuprofen behandelt wurde. Nach Auftreten der Psoriasis‐Erkrankung konnten die Gelenkbeschwerden auch im Zusammenhang mit der Hauterkrankung gesehen und als Psoriasis‐Arthritis interpretiert werden.

**ABBILDUNG 1:**
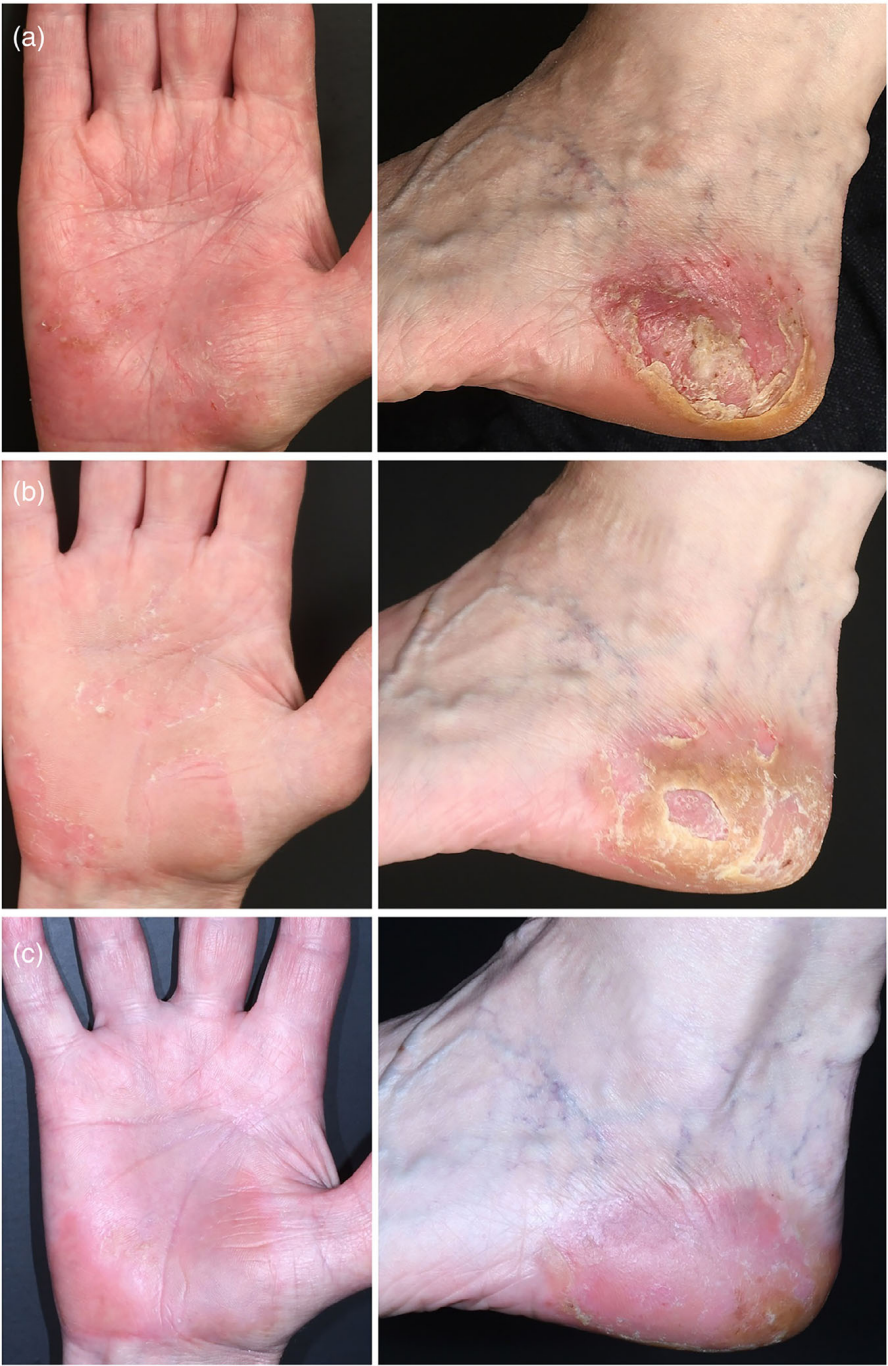
Befunde von (a) der rechten Handfläche und dem linken Fuß vor der Therapie mit Ruxolitinib, (b) nach 8 Wochen und (c) nach 10 Wochen unter Behandlung mit topischem Ruxolitinib.

Bei Aufnahme betrug der *Palmoplantar Pustulosis Area and Severity Index* (PPPASI) 6,2 von 72 (Abbildung 1a). Schmerz und Juckreiz wurden jeweils mit 9/10 auf der Numerischen Rating‐Skala (NRS) angegeben, der *Dermatology Life Quality Index* (DLQI) lag bei 17 von 30, was auf eine starke Beeinträchtigung der Lebensqualität hinwies. Aufgrund des unzureichenden Ansprechens auf konventionelle topische Therapien und der Ablehnung systemischer Behandlungen durch die Patientin erfolgte eine Off‐Label‐Therapie mit Ruxolitinib‐Creme (Opzelura™, 15 mg/g, zweimal täglich dünn aufgetragen). Nach dreiwöchiger Therapie hatten sich die Hautveränderungen gebessert und der PPPASI war auf 3,2 gesunken. Juckreiz, Schmerzen und Lebensqualität verbesserten sich ebenfalls (NRS 5/10 bzw. 3/10; DLQI 12). Um den therapeutischen Effekt zu verstärken, wurde das Medikament zweimal täglich für 1–2 Stunden unter einem Okklusivverband aus Frischhaltefolie appliziert. Nach weiteren 8 Wochen sank der PPPASI auf 2,0 (Abbildung 1b). Juckreiz, Schmerzen und Lebensqualität verbesserten sich ebenfalls weiter (NRS 5/10 bzw. 1/10; DLQI 7). Die Haut wurde unter der zweimal täglich durchgeführten Okklusivbehandlung sehr weich und empfindlich, so dass die Häufigkeit der Okklusivanwendung auf einmal täglich für 1–2 Stunden reduziert wurde. Nach weiteren 10 Wochen lag der PPPASI bei 0,8 (Abbildung 1c), Juckreiz und Schmerzen waren nicht mehr vorhanden (NRS 0/10 für beide Parameter). Es traten keine systemischen Nebenwirkungen auf. Die Patientin fühlte sich jedoch durch die mühsame Anwendung und die Zeit, welche die Creme zum Einziehen benötigte, etwas eingeschränkt.

Topische JAKi sind eine vielversprechende Klasse von Medikamenten zur Behandlung entzündlicher Hautkrankheiten. In der EU zugelassene topische JAKi sind der Pan‐JAKi Delgocitinib zur Behandlung von mittelschwerem bis schwerem chronischem Handekzem bei Erwachsenen^8^ und Ruxolitinib zur Behandlung von nichtsegmentaler Vitiligo mit Gesichtsbeteiligung bei Patienten ab 12 Jahren.^9^ Daten aus klinischen Studien zu topischem Ruxolitinib deuten auf eine Wirksamkeit bei atopischer Dermatitis, Vitiligo, Psoriasis und Lichen ruber hin.^10^ Ruxolitinib ist von der FDA (U.S. Food and Drug Administration) zur Behandlung leichter bis mittelschwerer atopischer Dermatitis bei Patienten ab 12 Jahren zugelassen.^11^ Eine pharmakokinetische Studie an Minischweinen zeigte, dass topisch appliziertes Ruxolitinib nur minimal systemisch resorbiert wurde und seine Konzentration in der Haut höher war als bei systemischer Anwendung.^12^

Klinische Studien sind notwendig, um die Wirkung von topischen JAKi wie Ruxolitinib bei der PPP zu bewerten.

## DANKSAGUNG

Open access Veröffentlichung ermöglicht und organisiert durch Projekt DEAL.

## INTERESSENKONFLIKT

R.M. war als Berater tätig und/oder erhielt Vortragshonorare und/oder Forschungsförderungen und/oder nahm an klinischen Studien der folgenden Firmen teil: AbbVie, Amgen, Almirall, Biogen IDEC, Boehringer‐Ingelheim, Celgene, Janssen‐Cilag, Leo, Lilly, Moonlake, MSD SHARP & DOHME, Novartis, Pfizer, UCB. M.P.S. war als Berater tätig und/oder erhielt Vortragshonorare und/oder Forschungsförderungen und/oder nahm an klinischen Studien der folgenden Firmen teil: AbbVie, Almirall, Biogen, Boehringer‐Ingelheim, BMS, Celltrion, Janssen‐Cilag, Leo, Lilly, Novartis, Scinai, UCB.

P.W. und N.C. geben keine Interessenkonflikte an.
